# Kaniwa (*Chenopodium pallidicaule*)’s Nutritional Composition and Its Applicability as an Elder-Friendly Food with Gelling Agents

**DOI:** 10.3390/gels9010061

**Published:** 2023-01-12

**Authors:** Dah-Sol Kim, Fumiko Iida

**Affiliations:** Department of Food and Nutrition, Japan Women’s University, Tokyo 112-8681, Japan

**Keywords:** Kaniwa, fatty acid, antioxidant, anti-diabetes, gelling agent, mousse, elder-friendly food

## Abstract

(1) Background: This study attempted to develop an elder-friendly food suitable to the Korean Industrial Standard (KS) after identifying the nutritional characteristics of Kaniwa; (2) Methods: The nutrient composition and physiological activity of Kaniwa were analyzed, and the concentration of the gelling agent (guar gum, locust bean gum, and xanthan gum) to be added to Kaniwa mousse was derived through regression analysis to suit KS hardness level 1 to 3; (3) Results: It was found that Kaniwa not only had a good fatty acid composition but also had good antioxidant and anti-diabetic properties. Moreover, it was found that in order to have the hardness to chew Kaniwa mousse with the tongue, it was necessary to add less than 1.97% guar gum, 4.03% locust bean gum, and 8.59% xanthan gum. In order to have a hardness that can be chewed with the gum, it was found that 2.17~4.97% guar gum, 4.45~10.28% locust bean gum, and 9.48~21.96% xanthan gum should be added; (4) Conclusions: As the aging rate and life expectancy increase, support for developmental research related to the elder-friendly industry should be continuously expanded in preparation for the upcoming super-aging society.

## 1. Introduction

Kaniwa (*Chenopodium pallidicaule*), also known as Cañihua or Cañahua, is a species of goosefoot native to the Andean region, cultivated as a pseudocereal crop for its seeds [1]. It is small in diameter, from 0.5 to 1.5 mm, brown or black in color, easily accessible, and drought-resistant, providing farmers with potential food and income [2]. Kaniwa can be easily milled into flour, which can be mixed with water and milk for breakfast [3]. In addition, it can also be used for many other purposes, such as making bread, pastry, and noodles. Alternatively, it can even be added to sweets, snacks, and weaning foods. Moreover, Kaniwa has distinct characteristics such as a high protein and dietary fiber content and abundant phenol content. Kaniwa’s protein, calcium, zinc, and iron content is higher than that of more widely commercialized cereals [4]. Lipids are mainly composed of unsaturated fatty acids (FA). In comparison with other cereals, Kaniwa is known for its low allergy risk—it does not exacerbate allergies. Moreover, the nutritional value of Kaniwa differs in the content of protein, which is 16% more in Kaniwa than in quinoa. Because it is gluten-free, it is known as a great choice for people with diabetes. Considering these characteristics, we thought Kaniwa would be suitable as an elder-friendly food material. This is because, in Korea, a country that has become a super-aging society, the demand for healthy foods containing a large amount of protein and essential fatty acids is increasing [5]. However, Kaniwa is now considered a forgotten crop that was once widely used in the Andes but is now replaced by other crops such as millet or quinoa [6]. Meanwhile, similar to the successful increase in demand for quinoa in western countries, Kaniwa also has growing market potential. Therefore, this study attempted to examine the possibility of using Kaniwa as an elder-friendly food.

The Ministry of Food and Rural Affairs of Korea established the Korean Industrial Standard (KS), an elder-friendly food certification system, in consideration of the increase in demand for elder-friendly foods that help the elderly consume, replenish, digest, and absorb nutrients [5]. This KS certification system sets quality standards for tooth intake (1st level; 55,000~500,000 N/m^2^), gum intake (2nd level; 22,000~50,000 N/m^2^), and tongue intake (3rd level; ≤20,000 N/m^2^), considering the hardness, viscosity, and nutrients of food, so the elderly can choose the appropriate food according to their health condition. It is also aimed at promoting the development of the elder-friendly food industry. Therefore, this study attempted to develop a mousse-type elder-friendly food that satisfies KS based on the nutritional characteristics of Kaniwa. Through this, it was intended not only to supplement nutrients such as protein and essential FAs needed by the elderly but also to improve the nutritional imbalance caused by reduced chewing ability due to aging.

## 2. Results and Discussion

### 2.1. Fatty Acid Composition

The total unsaturated FA content of Kaniwa was 76.90% (Table 1), which was found to be about three times higher than the total saturated FA content (23.10%). Eating foods high in unsaturated FAs is known to improve blood cholesterol levels, reduce the risk of stroke, ease inflammation, prevent blood clots, stabilize heart rhythms, maintain proper blood pressure, and play a number of other beneficial roles [7]. In addition, as it is known to be related to the cognitive function of the brain, it is actively encouraged for the elderly to consume foods containing a large amount of unsaturated FAs [8]. This is because the disease burden related to dementia, such as cognitive impairment caused by aging, is one of the major public health problems, considering Korea’s drastic demographic changes characterized by population aging. In particular, omega FAs, which are essential FAs, are known to play the most important role in brain function and normal growth and development, and since they are not synthesized in the body, they should be fully consumed through food.

Fortunately, linoleic acid (49.02%), which is omega-6, was found to be the most contained FA composition of Kaniwa. Omega-6 was reported to improve brain function in people with memory problems, such as those with Alzheimer’s disease or other cognitive impairments [8]. In addition, it also promotes skin and hair growth, maintains bone health, regulates metabolism, and helps maintain the reproductive system [9]. So, it is believed that Kaniwa can be used as an elder-friendly food material. This is because, in Korea, a country that has become a super-aging society, the elder-friendly food industry is developing to solve health problems such as cognitive decline and dementia, which are increasing in proportion to the rapidly increasing number of elderly people. In other words, the government is making great efforts to discover foods with high omega-6 content, and research on the development of elder-friendly foods using them is being actively conducted. According to Kim and Joo’s previous study [10], food with a high omega-6 content had a positive effect on the viability of hippocampus cells and brain neuron cells responsible for memory, and Korean elder-friendly foods were developed using this information. Next, Kaniwa was found to have a high oleic acid content (17.64%). It is chemically classified as a monounsaturated omega-9 FA and is the most abundant FA in human adipose tissue [11]. It is found in the phospholipids that make membranes, cholesterol esters, and wax esters and is known to help people suffering from mental and cognitive issues. Moreover, it was reported to support people with metabolic issues that may affect other parts of the body, including the brain. If omega-9 is deficient in the body, it is also necessary to ingest it from a diet because it induces a decrease in FAs in many tissues, including the brain [12]. Moreover, Kaniwa was found to contain 5.84% of α-linolenic acid, which is omega-3, and its deficiency in the body is known to alter the structure and function of the brain cell membrane and induce minor cerebral dysfunction. In addition, the elderly’s adequate intake (AIs) for omega-3 is set at 1.1 to 1.6 g, emphasizing the consumption of foods rich in omega-3 [13]. Considering these points, Kaniwa is likely to be used as an elder-friendly food material. Furthermore, it is thought that it can be helpful to provide a healthy diet with great nutritional quality to the elderly by developing elder-friendly foods using foods with high omega FAs, such as Kaniwa.

Meanwhile, unsaturated FAs such as omega-3, -6, and -9 have a shorter shelf-life than saturated FAs [14]. Moreover, unsaturated FAs also have lower oxidation stability than saturated FAs, which makes them more vulnerable to rancidity. This is due to the ability of double bonds to attract electrons from neighboring carbon, which makes hydrogen on the carbon more susceptible to abstraction, resulting in the formation of free radicals. Therefore, in addition to the discovery and development of foods with high essential FAs, as in this study, research on elder-friendly foods that improve the above limitations is expected to be conducted in the future.

### 2.2. Antioxidant Activity

#### 2.2.1. Total Polyphenol Content

The total polyphenol content of Kaniwa was 186.54 mg GAE/100 g (Table 2), similar to the results of studies on Kaniwa of Huamaní et al. (140~190 mg/100 g) and Repo-Carrasco-Valencia et al. (218~221 mg/100 g) [3,15]. It is believed that some differences are due to different varieties. Compared to other seeds, it was lower than buckwheat (323 mg/100 g), while it was higher than amaranth (21.2 mg/100 g), quinoa (71.7 mg/100 g), and wheat (53.1 mg/100 g) [16]. These plant-derived phenolics are considered the chief agents responsible for biological functioning and disease curing [17]. The epidemiological evidence indicates that a diet rich in plant-derived foods significantly reduces the risk of many types of cancer and cardiovascular disease, suggesting that certain dietary antioxidants could be effective agents in preventing cancer incidence and mortality. Plant-derived phenolics are also acknowledged as strong natural antioxidants that play a key role in a wide range of biological and pharmacological properties such as anti-inflammatory, anti-microbial, anti-allergic, anti-viral, anti-thrombotic, hepatoprotective, signaling molecules, and many more. As such, natural antioxidants are well known to have beneficial effects on humankind, and the discovery of new antioxidant foods offers prospects for innovation in the development of elder-friendly foods in addition to medicines. Therefore, this study on Kaniwa is thought to play an important role in the elder-friendly food industry.

#### 2.2.2. Total Flavonoid Content

The total flavonoid content of Kaniwa was 249.82 mg CAT/100 g, which was found to be similar to black rice (240.6 mg/100 g). Moreover, it was found to be higher than white rice (131.6 mg/100 g) and red rice (147.2 mg/100 g) [18]. These plant-derived flavonoids play a major protective role in oxidative stress conditions [17]. Numerous epidemiological and experimental evidence also exists to describe the protective role of flavonoids in degenerative diseases such as cardiovascular, cancer, diabetes, inflammation, and many more. Much of the literature also confirms that a flavonoid-rich diet provides better health for the elderly, mainly because it works as an antioxidant and inhibits oxidative damage-induced diseases. As such, natural antioxidants are well known to have beneficial effects on humanity, and the discovery of new antioxidant foods offers prospects for innovation in the development of elder-friendly foods. Therefore, this study on Kaniwa is thought to play an important role in the elder-friendly food industry.

#### 2.2.3. DPPH Radical Scavenging Activity

The DPPH radical scavenging activity of Kaniwa was 2.50 mg/mL, similar to the results of a study on Kaniwa by Huamaní et al. (1.3~4.2 mg/mL) [15]. It is believed that some differences are due to different varieties. However, it was higher than white jasmine rice (1.05 mg/mL), similar to red rice (2.29 mg/mL), and lower than quinoa (16.28~19.10 mg/mL) and black rice (5.93~6.93 mg/mL) [19,20]. As such, although the activity varies from food to food, it is found in various plant-based foods. Their health effects were recognized for their particularly strong antioxidant properties. Additionally, good DPPH radical scavenging activity was found to have diverse utility, including a role in nutrient uptake, structural components, enzyme activity, protein synthesis, photosynthesis, and allelopathy [17]. Meanwhile, for a better understanding of the health effects of plant-derived antioxidant activity in the elderly, a database on the possibility of using natural antioxidant foods such as Kaniwa as elder-friendly foods should be developed. This will provide a prospect for the elder-friendly food industry innovation.

#### 2.2.4. ABTS Radical Scavenging Activity

The ABTS radical scavenging activity of Kaniwa was 47.77 mg/mL: higher than red quinoa (40 mg/mL) while lower than white quinoa (110 mg/mL) [21]. The interest of the scientific community in this antioxidant activity continues to increase due to this antioxidant behavior and promising health benefits [17]. Such good natural antioxidant foods hinder the activity of the free radicals by numerous mechanisms and work against oxidative stress and its impediments by inhibiting the reactive oxygen species (ROS) producing enzymes. Related epidemiology evidence has shown that a diet with high consumption of fruits and vegetables with excellent antioxidant activity significantly reduces the risk of many cancers, suggesting that dietary antioxidants could be effective agents in preventing cancer incidence and mortality. Meanwhile, for a better understanding of the health effects of plant-derived antioxidant activity in the elderly, a database on the possibility of using natural antioxidant foods such as Kaniwa as elder-friendly foods should be developed. This will provide a prospect for the elder-friendly food industry innovation.

#### 2.2.5. Ferric Reducing Antioxidant Power

The FRAP of Kaniwa was 0.88 mol TE/100 g, lower than brown rice (1.45 mol/100 g), wheat (1.16~1.90 mol/100 g), corn (2.02~2.33 mol/100 g), oats (2.82~3.299 mol/100 g), rye (3.38 mol/100 g), and buckwheat (3.86 mol/100 g), while higher than barley (0.28 mol/100 g) and white rice (0.50 mol/100 g) [22]. As such, although the activity varies from food to food, it is found in various plant-based foods. Consumption of food based on such good antioxidant activity reduces the risk of disease and enhances human health [17]. Various health benefits and industrial applications of natural antioxidant foods such as Kaniwa draw the attention of the elder-friendly food-related industry to improve the quality of life through the nutritional balance of the elderly. Therefore, the discovery of new antioxidant foods will offer a prospect for innovation in the development of elder-friendly foods, and accordingly, this study on Kaniwa is thought to play an important role in the elder-friendly food industry.

#### 2.2.6. Superoxide Dismutase Activity

The SOD activity of Kaniwa was 193.20 U/g, which was found to be significantly lower than quinoa (500 U/g). Supplements are typically supplied as synthetic antioxidants, but recent studies confirmed that natural antioxidant foods such as vegetables, fruits, and grains provide more health benefits, such as preventing cell damage from free radical oxidation reactions, than antioxidant supplements, so there is a tremendous scope in their related industries [17]. Although Kaniwa showed lower SOD activity than quinoa in this study, plant-derived antioxidants are well known for playing a vital role in the prevention and treatment of cancer as they have a beneficial effect on the elderly. So the discovery of nutritional properties of natural foods such as Kaniwa will offer the possibility of innovation in the development of elder-friendly foods. Therefore, this study on Kaniwa is thought to play an important role in the elder-friendly food industry.

### 2.3. Antioxidant Activity

The α-amylase inhibitory activity of Kaniwa was 32.37 mg/mL, similar to the results of a study on Kaniwa (31.66 mg/mL) of Coronado-Olano, and higher than that of quinoa (11.18 mg/mL) [23]. In addition, the α-glucosidase inhibitory activity of Kaniwa was 7.84 mg/mL, indicating that Kaniwa had good antioxidant activity as well as anti-diabetic activity. Diabetes is identified as an oxidative stress disorder resulting from an imbalance between the formation of free radicals and the individual’s ability to oxidize them [17]. Oxidative stress is vastly associated with organ damage through ROS, which is poorly neutralized by antioxidants, resulting in inflammation and various metabolic disorders. On the other hand, antioxidants hinder the activity of free radicals by numerous mechanisms and work against oxidative stress and its impediments by inhibiting ROS-producing enzymes. Moreover, of all the properties shown by antioxidants, the best is the inhibition of α-glucosidase and α-amylase, which are accountable for converting dietary carbohydrates to glucose. Therefore, it is expected that Kaniwa’s good anti-diabetes ability is related to the antioxidant activity discussed above, and Kaniwa is thought to be able to be used as an elder-friendly food.

### 2.4. Hardness of Kaniwa Mousse

Poor chewing and swallowing can lead to difficulties in safely transporting food from the mouth to the esophagus, leading to dehydration, a nutritional deficit, aspiration pneumonia, and even death [24]. In particular, since chewing and swallowing functions deteriorate with age, they occur more often in the elderly. So, the use of rheology-modified food has become very important for the elderly with reduced chewing and swallowing function. Primarily, it has been reported that hardness, a force necessary for the deformation or penetration of food between the tongue and the palate, affects the elderly more than healthy adults. Therefore, this study aimed to determine how semi-solid food’s texture affects the effort of pharyngeal swallowing in the elderly over the age of 65. Therefore, this study attempted to provide the possibility that the elderly can easily chew according to different physical characteristics by preparing Kaniwa mousse in hardness stages 1st~3rd based on KS for elder-friendly foods. To this end, the effect on the hardness of Kaniwa mousse according to the concentration added for each gelling agent was investigated, and the results are shown in Table 3.

**Figure 1:**
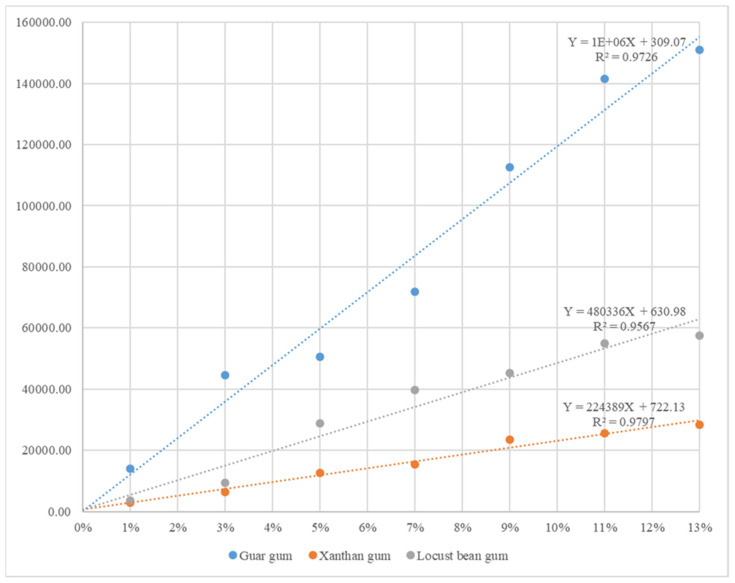
Regression equation for harness according to gelling agent of Kaniwa mousse. Y-scale is the “hardness (N/m^2^)” and X-scale is the “concentration (%) of the gelling agent”. The coefficient of determination (R^2^) is a number between 0 and 1 that shows how well the statistical model predicts the outcomes, and the higher the coefficient, the better the observations fit.

First of all, it may be a natural result, but as the concentration of all gelling agents increased, the hardness significantly increased. Moreover, there was also a significant difference between the gelling agents at each same concentration. Guar gum showed the greatest change in hardness, showing a hardness that can be chewed with the tongue (KS 3rd) when added 1% is added, a hardness that can be chewed with the gum (KS 2nd) when 3~5% is added, and a hardness that can be chewed with the teeth (KS 1st) when 7~13% is added. Guar gum, also called guaran, is a galactomannan polysaccharide extracted from guar beans that has thickening and stabilizing properties useful in food applications [25]. Moreover, its low digestibility lends its use in recipes as a filler, which can help to provide satiety or slow the digestion of a meal, thus lowering the glycemic index of that meal. In the case of locust gum, we found a hardness that can be chewed with the tongue when 1~3% is added, a hardness that can be chewed with the gums when 5~9% is added, and a hardness that can be chewed with the teeth when 11~13% is added. Locust bean gum consists chiefly of high-molecular-weight hydrocolloidal polysaccharides composed of galactose and mannose units combined through glycosidic linkages, which may be described chemically as galactomannan [26]. It has a sweet flavor similar to chocolate, so it is used to sweeten foods and as a chocolate substitute. It is also used in pet foods and inedible products such as mining products, paper making, and as a thickener in textiles. In the case of xanthan gum, we found a hardness that can be chewed with the tongue when 1~7% is added and a hardness that can be chewed with the gums when 9~13% is added, resulting in the slightest change in hardness among these three gelling agents. Xanthan gum is composed of pentasaccharide repeat units comprising glucose, mannose, and glucuronic acid in the molar ratio of 2:2:1 [27]. In foods, xanthan gum is common in salad dressings and sauces [28]. It helps to prevent oil separation by stabilizing the emulsion, although it is not an emulsifier. Xanthan gum also helps create the desired texture in many ice creams. It is also a preferred method of thickening liquids for those with swallowing disorders since it does not change the color or flavor of foods or beverages at typical use levels. Based on these results, regression analysis was performed to investigate the relationship between the dependent variable (hardness) and the independent variable (type of gelling agent and its added concentration), and the regression equation was obtained with high accuracy as follows: “Y = 1E + 06X + 309.07 (R^2^ = 9726)” for guar gum, “Y = 480336X + 630.98 (R^2^ = 9567)” for locust bean gum, and “Y = 224,389X + 722.13 (R^2^ = 0.9797)” for xanthan gum (Figure 1). Through these regression equations, it was found that in order to have the hardness to chew Kaniwa mousse with the tongue, it was necessary to add less than 1.97% guar gum, 4.03% locust bean gum, and 8.59% xanthan gum. In order to have a hardness that can be chewed with the gum, it was found that 2.17~4.97% guar gum, 4.45~10.28% locust bean gum, and 9.48~21.96% xanthan gum should be added. Moreover, in order to have a hardness that can be chewed with teeth, it was found that it was necessary to add at least 5.47% guar gum, 11.32% locust bean gum, and 24.19% xanthan gum. Considering that the elderly have different physical characteristics, these results are expected to provide health benefits by providing elder-friendly foods for each characteristic group [29]. In particular, guar gum can greatly change its rheological properties with a small amount, so it is expected to bring economic benefits to the elder-friendly food industry. In other words, all three gelling agents used in this study were found to make mousse-type food suitable for KS, especially guar gum, which is believed to have the greatest economic advantage for the elder-friendly food industry among the three, as it is easy to adjust hardness even with a small amount.

## 3. Conclusions

After identifying the nutritional characteristics of Kaniwa, this study attempted to develop a mousse-type elder-friendly food using Kaniwa suitable for KS in consideration of the elderly whose chewing function deteriorated due to the deterioration of muscles, such as the oral and esophageal muscles, due to aging. Through this, it was intended to promote a healthy quality of life by helping improve health problems such as nutritional imbalance and cognitive decline in the elderly. As a result, Kaniwa had a high content of omega FAs and had good antioxidant and anti-diabetic activity. Through these, it was confirmed that Kaniwa could be sufficiently used as an elder-friendly food material. So, we developed a mousse-type elder-friendly food using Kaniwa, and it was also confirmed that the gelling agent used for this could be used in the development of elder-friendly food suitable for KS. Specifically, it was found that in order to have a hardness to chew Kaniwa mousse with the tongue, it was necessary to add less than 1.97% guar gum, 4.03% locust bean gum, and 8.59% xanthan gum. In order to have a hardness that can be chewed with the gum, it was found that 2.17~4.97% guar gum, 4.45~10.28% locust bean gum, and 9.48~21.96% xanthan gum should be added. Moreover, in order to have a hardness that can be chewed with teeth, it was found that it was necessary to add at least 5.47% guar gum, 11.32% locust bean gum, and 24.19% xanthan gum. As such, considering that the elderly have different physical characteristics, it is expected that risk factors caused by poor chewing function can be prevented by providing elder-friendly foods suitable for each characteristic group. In conclusion, as the aging rate and life expectancy increase, the size of the elder-friendly food industry will continue to grow, and support for developmental research related to the elder-friendly industry should be continuously expanded in preparation for the upcoming super-aging society. Meanwhile, the limitations of this study are not only the analysis of the nutritional characteristics of food materials such as Kaniwa but also the health functionality of the finally developed elder-friendly foods (such as the Kaniwa mousse developed in this study), and they should be studied in the future.

## 4. Materials and Methods

### 4.1. Sample Preparation

Raw Kaniwa (5 kg cultivated in Peru) was purchased from Yupik Co. (Montreal, QC, Canada) and used as a sample for physicochemical experiments (fatty acid composition, antioxidant, and anti-diabetic activity).

### 4.2. Analysis of Fatty Acid Composition

The FA composition of fat fraction was determined after methylation according to the procedure described by Repo-Carrasco-Velncia [30]. Of the sample, 1 g was put into a 50 mL test tube, 2 mL of a 5% pyrogallol ethanol solution was added, and 1 mL of triundecanoin was added. Of 8.3 M hydrochloric acid, 10 mL was added, mixed with a vortex mixer for 30 s, and then decomposed for 60 min while stirring at 200 rpm at 80 °C, and then cooled to room temperature. After adding 15 mL of diethyl ether and mixing it with vortex mixer for 30 s, the supernatant was collected in a new 50 mL test tube by passing a syringe filled with 5 g of Na_2_SO_4_. Of petroleum ether, 15 mL was added, mixed with vortex mixer for 30 s, and the supernatant was again passed through Na_2_SO_4_ to be combined into a 50 mL test tube. The solvent collected in the test tube was evaporated and removed at 40 °C under a nitrogen stream, and the crude fat of the sample was extracted. Of a 0.5 N methanolic sodium hydroxide solution, 1.5 mL was added to the extracted crude fat, mixed with a vortex mixer, and quenched in an 85 °C water bath for 10 min. After cooling this, 2 mL of a 14% BF_3_ methanol solution was added and mixed and then quenched in an 85 °C water bath for 10 min to manufacture FA methyl ester (FAME). This was cooled to room temperature, and 2 mL of isooctane and 1 mL of saturated NaCl solution were added, mixed, and then cooled to room temperature. Its supernatant was analyzed using a gas chromatograph (6890N GC-FID, Agilent, Santa Clara, CA, USA) as a test solution by passing through a fused silica capillary column (Omegawax^TM^ 250, L × I.D. 30 m × 0.25 mm, df 0.25 μm, Supelco, Bellefonte, PA, USA). The initial oven temperature was maintained at 160 °C for 1 min, raised to 240 °C at a rate of 4 °C/min, and then maintained for 5 min. The injector and detector temperatures were set at 240 °C and 260 °C, respectively. Helium at a flow rate of 1.1 mL/min was used as the carrier gas. The split ratio was set at a ratio of 200:1. Identification of FAME was performed by comparing the standard (SMB00937, Sigma-Aldrich, Bellefonte, PA, USA) with the retention time. The results were expressed as a percentage of the total FAME analyzed. This experiment was repeated three times.

### 4.3. Analysis of Antioxidant Activity

All antioxidant activity experiments were repeated three times.

#### 4.3.1. Total Polyphenol Content

The total polyphenol content was determined by Folin–Ciocalteu’s method, according to Huamani et al. [15]. Of the sample, 2 g was added to 15 mL of 80% ethanol and extracted at 37 °C for 18 h. The extract was centrifuged at 5000 rpm at 4 °C for 15 min to measure the supernatant with a final volume of 25 mL in a volumetric flask. Of aliquot, 50 μL was mixed with 1 mL of Folin–Ciocalteu reagent diluted with distilled water (1:10, *v:v*). The mixture was pre-incubated for 2 min, and then 1 mL of a Na_2_CO_3_ solution (7.5%) was added to it and incubated in the dark at room temperature for 15 min. The absorbance (Abs) of the reaction mixture was measured at 750 nm using a spectrophotometer (T60, PG instruments Ltd., Lutterworth, UK). The total polyphenol content of the sample was calculated using a gallic acid calibration curve and was expressed as mg of the gallic acid equivalent (GAE) per 100 g of the sample.

#### 4.3.2. Total Flavonoid Content

The total flavonoid content was determined according to the Huamani et al. method [15]. Of the sample, 2 g was extracted at 37 °C with 20 mL of 80% ethanol for 18 h, filtered, and then measured in a volumetric flask to have a final volume of 25 mL. Of the extract, 0.5 mL was mixed with 1.5 mL distilled water and 0.15 mL NaNO_2_ solution (0.05%) and left at room temperature for 5 min. Thereafter, 0.15 mL of a 0.1% AlCl_3_ solution was added to the mixture, left at room temperature for 6 min, and then 1 mL of 1M NaOH was added. After adding distilled water to make the total volume of the reaction mixture 5 mL, the Abs thereof was measured at 510 nm using a spectrophotometer (T60, PG instruments Ltd., Lutterworth, UK). The total flavonoid content of the sample was calculated using a catechin calibration curve and was expressed as mg of the catechin equivalent (CAT) per 100 g of the sample.

#### 4.3.3. DPPH Radical Scavenging Activity

The antioxidant activity was determined by Huamani et al.’s method [15]. Of the sample, 1 g was extracted at 37 °C with 5 mL of 80% ethanol for 18 h, filtered, and then measured in a volumetric flask to have a final volume of 25 mL. Of aliquot, 50 μL was mixed with 1 mL of 0.1 mM 2,2-diphenyl-1-picrylhydrazyl (DPPH) and left for 30 min in a dark place at room temperature. Thereafter, the Abs was read using 80% ethanol as a control with a spectrophotometer (T60, PG instruments Ltd., Lutterworth, UK) at 517 nm. The DPPH radical scavenging activity of the sample was calculated according to the following formula: Inhibition (%) = [100 × {(Abs_control_ − Abs_sample_)/Abs_control_}]. DPPH radical scavenging activity was expressed as FSC_50_ value (extract concentration (mg/mL) that provided 50% of the activity based on the dose-dependent mode of action).

#### 4.3.4. ABTS Radical Scavenging Activity

The antioxidant activity was determined by the method of Złotek et al. as the ability to scavenge free ABTS^•+^ radicals [21]. The ABTS solution was prepared by mixing 7 mM ABTS and 2.45 mM potassium per sulfate (1:1, *v:v*), leaving the mixture in a dark place at room temperature for 16 h and diluting it with distilled water so that the absorbance measurement at 734 nm was 0.70~0.72. Of the sample, 1 g was extracted at 37 °C with 5 mL of 80% ethanol for 18 h, filtered, and then 0.04 mL of the extract and 1.8 mL of ABTS solution were mixed. The Abs of the mixture was measured in even minutes of reaction during 10 min at 734 nm. Moreover, Abs was read with a spectrophotometer (T60, PG instruments Ltd., Lutterworth, UK) using an ABTS solution as a control. The ABTS radical scavenging activity of the sample was calculated according to the following formula: Inhibition (%) = [100 × {(Abs_control_ − Abs_sample_)/Abs_control_}]. ABTS radical scavenging activity was expressed as EC_50_ value (extract concentration (mg/mL) that provided 50% of the activity based on the dose-dependent mode of action).

#### 4.3.5. Ferric-Reducing Antioxidant Power

The ferric-reducing antioxidant power (FRAP) was determined by Peñarrieta et al.’s method [31]. FRAP reagent was prepared by mixing 0.1 mol/L sodium acetate buffer (pH 3.6), 10 mmol/L TPTZ, and 20 mmol/L ferric chloride (10:1:1, *v:v:v*) [31]. Of the sample, 1 g was extracted at 37 °C with 5 mL of 80% ethanol for 18 h, filtered, and then 30 µL of the extract, 900 µL of the FRAP reagent, and 90 µL of distilled water were mixed. Measurement of the Abs of the mixture was performed for 10 min every 20 s, starting immediately after the addition of the extract at 593 nm with a spectrophotometer (T60, PG instruments Ltd., Lutterworth, UK). Moreover, the control consists of 120 µL of distilled water and 900 µL of FRAP reagent. The FRAP of the sample was calculated using a Trolox calibration curve and was expressed as mol of the Trolox equivalent (TE) per 100 g of the sample.

#### 4.3.6. Superoxide Dismutase Activity

Superoxide dismutase (SOD) activity was measured by the enzyme’s ability to inhibit photoreduction of nitroblue tetrazolium (NBT) [32]. Of the sample, 2 g was added to 15 mL of 80% ethanol, extracted at 37 °C for 18 h, filtered, and 10 μL of aliquot of it was mixed with the incubation medium solution (a mixture of 100 µL of 100 mM potassium phosphate (pH 7.8), 40 µL of 70 mM methionine, 3 µL 10 µM EDTA, 31 µL of distilled water, 15 µL of 1 mM NBT, and 2 µL of 0.2 mM riboflavin). This was illuminated for 7 min with a 20 W fluorescent lamp. Moreover, the same medium solution without an extract was used as a control. The Abs was measured at 560 nm with a spectrophotometer (T60, PG instruments Ltd., Lutterworth, UK), and the SOD activity of the sample was calculated according to the following formula: Inhibition (%) = [100 × {Abs_sample_ − Abs_control_)/Abs_control_}]. The SOD activity was expressed in U per g of the sample (one unit of the SOD can inhibit 50% of the photoreduction of the NBT under assay conditions).

### 4.4. Analysis of Anti-diabetic Activity

All anti-diabetic activity experiments were repeated three times.

#### 4.4.1. α-Amylase Inhibitory Activity

The inhibitory activity of α-amylase was determined by Coronado-Olano et al.’s method [23]. Of the sample, 2 g was added to 15 mL of 80% ethanol, extracted at 37 °C for 18 h, filtered, and then 50 µL of the extract was mixed with 50 µL of α-amylase pancreatic enzyme solution dissolved in 0.1 M phosphate-buffered saline (pH 6.9) at a concentration of 5 U/mL. After the mixture was pre-incubated at 37 °C for 60 min, 50 µL of a starch solution (0.5% *w/v*) dissolved in 0.1 M phosphate buffered saline was added and then incubated at 37 °C for 5 min. Thereafter, 50 µL of 96 mM dinitrosalicylic acid was added, incubated in a water bath at 100 °C for 5 min, and cooled for 5 min at room temperature. The Abs of the reaction mixture was read at 540 nm with a spectrophotometer (T60, PG instruments Ltd., Lutterworth, UK), and the percentage of α-amylase inhibition was calculated using following formula: Inhibition (%) = [100 × {(Abs_control_ − Abs_sample_)/Abs_sample_}]. α-Amylase inhibitory activity was expressed as IC_50_ value (concentration (mg/mL) required to obtain 50% inhibition of enzyme activity).

#### 4.4.2. α-Glucosidase Inhibitory Activity

The inhibitory activity on α-glucosidase was determined by Coronado-Olano et al.’s method [23]. Of the sample, 2 g was added to 15 mL of 80% ethanol, extracted at 37 °C for 18 h, filtered, and then 50 µL of 0.1 M potassium phosphate buffer (pH 6.9) and 100 µL of 0.1 M potassium phosphate buffer (pH 6.9) containing α-glucosidase solution (0.5 U/mL) of saccharomyces cerevisiae were mixed with 50 µL of the extract. After pre-incubating the mixture at 37 °C for 30 min, 50 µL of a 5 mM solution of p-nitrophenyl-D-α-glucopyranoside in 0.1 M potassium phosphate buffer (pH 6.9) was added and incubated at 37 °C for 5 min. Of sodium carbonate (Na_2_CO_3_), 50 µL was added to stop the reaction of the mixture and incubated at 37 °C for 5 min, and then its Abs was read at 405 nm with a spectrophotometer (T60, PG instruments Ltd., Lutterworth, UK). The control contained 50 µL of a buffer solution instead of the extract, and the change in Abs before and after incubation of the control and the experimental group was measured. The percentage of α- glucosidase inhibition was calculated using following formula: Inhibition (%) = [100 × {(Abs_control_ − Abs_sample_)/Abs_sample_}]. α-Glucosidase inhibitory activity was expressed as IC_50_ value (concentration (mg/mL) required to obtain 50% inhibition of enzyme activity).

### 4.5. Manufacturing of Elder-Friendly Food

Moreover, for the development of mousse-type elder-friendly food using Kaniwa, it was prepared as follows [33]. Grind 12 tablespoons of raw Kaniwa and simmer at 60 °C for about 15 min with 4 cups of milk, then add 2 tablespoons of sugar, 2 teaspoons of vanilla extract, and each gelling agent (guar gum, locust bean gum, and xanthan gum; concentration of 1~13% of total weight) and boil at 80 °C for another 2 min. This was placed in a circular mold with a diameter of 5 cm and a height of 2.5 cm and left in the refrigerator for 2 h. It was used as an experimental group for texture profile analysis (TPA).

### 4.6. Hardness

TPA was performed using a texture analyzer (TA-XT Express 20096, Stable microsystems Ltd., London, UK), and a two-cycle compression test was performed using a 25 kg load cell. In addition, a 10 mm diameter cylindrical probe (pre-test speed 3 mm/s, trigger force 5 g, test speed 3 mm/s, return speed 3 mm/s, test distance 5 mm, time 5 s) was used to compress the Kaniwa mousse, and the TPA recorded hardness. This experiment was repeated three times.

### 4.7. Statistical Analysis

The results of this experiment were tested using a one-way analysis of variance (ANOVA). If the test results were significant, the test was performed using Duncan’s post-hoc test to analyze significant differences between the test groups. These statistical analyses used IBM SPSS statistics (Version 23.0, GraphPad Software Inc., San Diego, CA, USA) and were determined to be statistically significant if the *p*-value was less than 0.05 (*p* < 0.05).

## Figures and Tables

**Table 1:** Fatty acid composition of Kaniwa.

	Fatty Acid	Content (%) ^1^
Saturated fatty acid	Myristic acid (C_14:0_)	0.74 ± 0.00
	Pentadecylic acid (C_15:0_)	0.19 ± 0.00
	Palmitic acid (C_16:0_)	15.77 ± 0.35
	Margaric acid (C_17:0_)	0.10 ± 0.00
	Stearic acid (C_18:0_)	3.21 ± 0.15
	Arachidic acid (C_20:0_)	1.02 ± 0.03
	Behenic acid (C_22:0_)	1.49 ± 0.06
	Tricosanoic acid (C_23:0_)	0.03 ± 0.00
	Lignoceric acid (C_24:0_)	0.56 ± 0.03
	Total	23.10 ± 0.62
Unsaturated fatty acid	Palmitoleic acid (C_16:1_)	0.96 ± 0.00
	Heptadecenoic acid (C_17:1_)	0.11 ± 0.00
	Vaccenic acid (C_18:1n7c_)	0.47 ± 0.01
	Oleic acid (C_18:1n9c_)	17.64 ± 0.19
	Linoleic acid (C_18:2n6_)	49.02 ± 0.77
	α-Linolenic acid (C_18:3n3_)	5.84 ± 0.00
	Eicosenoic acid (C_20:1n9_)	1.63 ± 0.05
	Eicosadienoic acid (C_20:2n6_)	0.05 ± 0.00
	Erucic acid (C_22:1n9_)	1.18 ± 0.01
	Total	76.90 ± 1.05
Total fat		6.09 ± 0.23

^1^ The results were expressed as mean ± standard deviation of the experiment repeated three times.

**Table 2:** Antioxidant and anti-diabetic activity of Kaniwa.

	Physiological Activity	Status ^1^
Antioxidant activity	Total polyphenol content (mg GAE/100 g)	186.54 ± 8.97
	Total flavonoid content (mg CAT/100 g)	249.82 ± 7.45
	FSC_50_ of DPPH radical scavenging activity (mg/mL)	2.50 ± 0.11
	EC_50_ of ABTS radical scavenging activity (mg/mL)	47.77 ± 2.13
	Ferric reducing antioxidant power (mol TE/100 g)	0.88 ± 0.04
	Superoxide dismutase activity (U/g)	193.20 ± 3.50
Anti-diabetic activity	IC_50_ of α-amylase inhibitory activity (mg/mL)	32.37 ± 1.52
	IC_50_ of α-glucosidase inhibitory activity (mg/mL)	7.84 ± 0.29

^1^ The results were expressed as mean ± standard deviation of the experiment repeated three times.

**Table 3:** Hardness of Kaniwa mousse according to gelling agent.

Concentration (%)	Hardness (N/m^2^) ^1^			F-Value ^2^ (*p*-Value)
	Guar Gum	Locust Bean Gum	Xanthan Gum	
1	13,953.67 ± 664.29 ^fA^	3730.33 ± 72.46 ^fB^	2878.67 ± 144.92 ^gC^	731.169 *** (0.000)
3	44,479.00 ± 1284.08 ^eA^	9480.67 ± 245.08 ^eB^	6417.00 ± 298.68 ^fC^	2238.155 *** (0.000)
5	50,600.00 ± 2455.20 ^eA^	28,940.33 ± 1240.83 ^dB^	12,738.67 ± 204.53 ^eC^	426.793 *** (0.000)
7	71,805.67 ± 3142.66 ^dA^	39,700.00 ± 1473.09 ^cB^	15,471.33 ± 761.10 ^dC^	569.243 *** (0.000)
9	112,701.67 ± 5563.86 ^cA^	45,338.00 ± 2019.85 ^bB^	23,500.00 ± 1000.00 ^cC^	539.943 *** (0.000)
11	141,433.33 ± 6880.65 ^bA^	55,033.33 ± 2000.83 ^aB^	25,633.33 ± 1184.62 ^bC^	618.169 *** (0.000)
13	151,033.33 ± 4528.06 ^aA^	57,558.67 ± 2796.59 ^aB^	28,366.67 ± 1096.97 ^aC^	1251.547 *** (0.000)
F-value (*p*-value)	489.888 *** (0.000)	480.394 *** (0.000)	464.420 *** (0.000)	

^1^ The results were expressed as mean ± standard deviation of the experiment repeated three times. ^2^ One-way ANOVA was used, and different letters in the same row (A~C) and column (a~g) mean a significant difference at *** *p* < 0.001.

## Data Availability

Not applicable.
